# Synthesis of Magnetic Nanoparticle/Polymer Matrix Nanocomposites with Induced Magnetic Performance

**DOI:** 10.3390/polym17141913

**Published:** 2025-07-10

**Authors:** Anastasios C. Patsidis, Aikaterini Sanida, Georgia C. Manika, Sevasti Gioti, Georgios N. Mathioudakis, Nicholas Petropoulos, Athanasios Kanapitsas, Christos Tsonos, Thanassis Speliotis, Georgios C. Psarras

**Affiliations:** 1 Smart Materials & Nanodielectrics Laboratory, Department of Materials Science, School of Natural Sciences, University of Patras, 26504 Patras, Greece or sanida.1@osu.edu (A.S.); manikage@upatras.gr (G.C.M.); s.gioti@upnet.gr (S.G.); 2 William G. Lowrie Department of Chemical & Biomolecular Engineering, The Ohio State University, 151 W. Woodruff Avenue, Columbus, OH 43210, USA; 3 Institute of Chemical Engineering Sciences (ICE-HT), Foundation for Research & Technology-Hellas (FORTH), Stadiou Str., Platani, 26504 Patras, Greece; mathioy@iceht.forth.gr; 4 Department of Physics, University of Thessaly, 35100 Lamia, Greece; npetropoulos@uth.gr (N.P.); kanapitsas@uth.gr (A.K.); christostsonos@uth.gr (C.T.); 5 Institute of Nanoscience and Nanotechnology, NCSR “Demokritos”, Aghia Paraskevi, 15310 Athens, Greece; t.speliotis@inn.demokritos.gr

**Keywords:** hybrid nanocomposites, polymer matrix nanocomposites, magnetic response, magnetic nanocomposites, magnetic susceptibility

## Abstract

In this work magnetic nanoparticles (Fe_3_O_4_, or ZnFe_2_O_4_, or SrFe_12_O_19_) and BaTiO_3_ microparticles were embedded in an epoxy resin for the synthesis of three series of hybrid magnetic polymer nanocomposites. Barium titanate content was kept constant, while magnetic phase content was varied. Fabricated specimens were structurally and morphologically characterized by employing scanning electron microscopy images and X-ray diffraction patterns. Results implied successful synthesis of the hybrid nanocomposites. The magnetic behavior of the pure magnetic nanoparticles and the fabricated nanocomposites was investigated via a Vibrating Sample Magnetometer. The magnetic performance of each type of magnetic phase (i.e., soft and hard) was induced in the nanocomposites, and magnetic performance is strengthened with the increase in magnetic phase content. Initial magnetization curves were used for the determination of mass magnetic susceptibility of all nanocomposites. Magnetic saturation and magnetic remanence have been found to follow a linear relationship with magnetic phase content, giving the opportunity to predict the system’s response in advance.

## 1. Introduction

Nowadays, polymer matrix nanocomposites (PNCs) are considered an important class of engineering materials for various technological applications. Epoxy resins are the most popular matrices for the development of advanced composites since they are characterized by low weight, processing easiness, flexibility, good wetting, and adhesion with the majority of employed fillers at a low cost. Their properties can be improved by integrating various fillers, which advance their thermomechanical properties, dielectric and electrical responses, magnetic properties, and the ability to store and recover electrical energy. By these means, multifunctional performance can be induced by integrating suitable fillers, which exhibit supplementary properties/responses [1,2,3]. Multifunctional performance or equivalently multiresponsive materials’ systems can be defined as the set of various desirable and tuned properties, which constitute a material’s behavior under the influence of suitable stimuli or a control signal [3,4,5].

Research focus for PNCs remains on their thermomechanical properties and partially on their electrical properties, even if the employed reinforcing phase is a magnetic substance such as iron oxide or a ferrite [6,7,8,9,10,11,12]. However, nanocomposites incorporating magnetic nanoparticles (MNPs) into polymer matrices introduce an additional aspect in their performance. Among mechanical, thermal, and electrical properties, the magnetic response further completes the multifunctional performance of PNCs. Integrating MNPs into a polymer matrix opens novel routes for applications in the fields of electromagnetic interference (EMI) shielding, smart coatings, information technology, telecommunication, damage detection techniques, magnetic resonance imaging, biomedical devices, and advanced structural materials.

Until now, research studies of the magnetic properties of PNCs in the literature are limited, and the majority refer to binary systems [13,14,15,16]. Previous studies on PNCs with embedded MNPs attempt to exploit in the same material the dielectric behavior resulting from the matrix and filler and the magnetic properties resulting from the reinforcing phase [13,14,15,16]. Iron oxide and various ferrites are semiconducting magnetic materials, thus affecting both the electrical and magnetic performance of PNCs when employed in a polymer matrix. Even in the case of employing metal nanoparticles such as Ni or Fe in a polymer matrix, the dielectric response of the composites is enhanced because of the conductivity of the isolated inclusions and the formed interfaces [13]. The magnetic performance of PNCs follows that of employed MNPs. A deeper analysis in the case of Fe_3_O_4_/epoxy PΝCs, conducted via a superconducting quantum interference device (SQUID) magnetometer, revealed two transitions at 90 and 30 K [17]. The maximum value of the applied field was 50 kOe, while the temperature range varied between 5 and 300 K. The zero-field-cooled (ZFC) and field-cooled (FC) processes gave evidence of these transitions, which were attributed to the Verwey transition (the first one) and to a spin-glass-like transition (the second one). The Verwey transition is a first-order magnetic phase transition due to variation in magnetocrystalline anisotropy influencing the magnetic alignment of spins. The spin-glass transition represents a change from the ferrimagnetic state at a high temperature to the paramagnetic state at a low temperature and occurs at the grains’ surfaces [17]. Other studies refer to the possible presence of α-Fe_2_O_3_, which in its bulk form undergoes a magnetic transition from a weak ferromagnetic phase to an antiferromagnetic phase. This transition is recorded in the vicinity of 260 K and is known as the Morin transition. The temperature where this transition occurs is affected by the particles’ shape and size, and crystallinity [18,19,20]. The magnetic performance of hybrid or ternary PNCs, which employ two different reinforcing phases, has not been studied extensively and in a comparable way between the various fillers.

The present work is part of a wider project where different MNPs are employed in an epoxy resin together with barium titanate particles, aiming to utilize the properties of all three constituents [4,5]. In this study, a series of hybrid epoxy nanocomposites with magnetic nanoparticles (Fe_3_O_4_, or ZnFe_2_O_4_, or SrFe_12_O_19_) and BaTiO_3_ have been prepared and studied. The fabricated hybrid systems could respond under various loading conditions, exhibiting suitable mechanical/thermal properties, tunable electric conductivity, variable electric polarization/dielectric permittivity, thermally induced phase changes, and an adjustable magnetic response. Barium titanate has been chosen for its dielectric and functional properties [4,5]. However, the subject of the present work is the induced magnetic performance on hybrid PNCs by embedded MNPs. Barium titanate is a well-known ceramic ferroelectric material, characterized by high dielectric permittivity, transition from the polar ferroelectric phase (tetragonal crystal structure) to the non-polar paraelectric phase (cubic crystal structure) at a critical temperature (Curie temperature, T_C_~130 °C), and piezoelectric properties [21,22,23,24,25,26,27]. By these means, the use of BaTiO_3_ as a filler in a polymer matrix enhances the dielectric response of the composites and induces functional behavior because of its variable polarization, piezoelectric properties, and the ability to store and retrieve electrical energy [28,29,30]. It should be noted that the switching behavior of BaTiO_3_ between the ferroelectric and paraelectric phases lowers by reducing the dimensions of the ceramic particles. Tetragonality expresses the aspect ratio (a/c) between the edge of the square base and the height of the tetragonal unit cell. By reducing the dimensions of the unit cell, at the nanoscale level, the ratio a/c tends to unity (a/c → 1) and the crystal structure approaches the cubic one even below T_C_ [22,23,24,25,26,27]. Thus, the ability to transform from polar to non-polar status diminishes. It has been found that at the nanoscale level and below the critical temperature, in barium titanate particles both cubic and tetragonal phases co-exist [21,22,23,24,25,26,27]. Aiming to keep the functional behavior of barium titanate in fabricated PNCs, the employed ceramic particles were at the microscale. Moreover, the amount of the integrated barium titanate in the hybrid PNCs was dictated by previous studies from our group [26,31,32].

According to the narrow or wide hysteresis curves of magnetization versus magnetic field, magnetic materials are classified into soft and hard magnets. In addition, ordering of the atomic magnetic moments, in the absence of a magnetic field, classifies magnetic materials as follows: (i) ferromagnetic (with equal magnetic moments aligned in the same direction), (ii) antiferromagnetic (with antiparallel equal magnetic moments), and (iii) ferrimagnetic (with antiparallel unequal magnetic moments). In the present study, the employed magnetic nanoparticles correspond to a typical magnetic material (Fe_3_O_4_) that was found to exhibit soft behavior, and a hard (SrFe_12_O_19_) and a superparamagnetic (ZnFe_2_O_4_) ferrite.

Magnetite (Fe_3_O_4_) is probably the most common iron oxide in nature and is characterized by the strongest magnetic properties between the transition metal oxides [33]. In its crystal structure, which is an inverse spinel, Fe^3+^ ions are randomly distributed between the octahedral and tetrahedral sites, while Fe^2+^ ions are placed at octahedral lattice sites [34,35]. At room temperature magnetite exhibits ferrimagnetic behavior with a Curie temperature close to 577 °C. Fe_3_O_4_ particles at the nanoscale level are usually single-domain magnetic materials, with narrow or even no hysteresis loop. Under this condition they display a superparamagnetic response [33,36]. Magnetite is exploited in numerus technological applications, including biomedical devices, drug delivery, magnetic resonance imaging, information storing media, catalysts, magnetic fluids, etc [21,33,34,35,36,37,38]. Ferrites represent an interesting class of magnetic materials either with soft or hard magnetic behavior. Their crystal structure could be garnet, spinel, or hexagonal [34]. Zinc ferrite (ZnFe_2_O_4_) crystallizes in a normal spinel structure with the Zn^2+^ ions occupying the tetrahedral sites and Fe^3+^ ions the octahedral ones. Typically, ZnFe_2_O_4_ is considered an antiferromagnetic material with paramagnetic behavior at room temperature. However, deviations from this behavior have been reported for nanosized ZnFe_2_O_4_ particles [39,40,41,42,43]. Zinc ferrite displays moderate saturation magnetization, high electrical resistivity, and low eddy current losses, and is exploited in applications such as sensors, microwave devices, data storage, gyrators, and information delivery devices [34,35,37]. Strontium ferrite (SrFe_12_O_19_) is a hexagonal ferromagnetic material. Hexaferrites exhibit enhanced magneto-crystalline anisotropy, saturation magnetization, intrinsic coercivity, and electrical resistivity [35,44]. Hexagonal ferrites are used as permanent magnets, founding applications in magnetic recording technology, high-frequency electronics, microwave-based devices, and in the field of electrical power production and distribution [35,37,44,45,46].

The prepared series of nanocomposites were morphologically and structurally examined via scanning electron microscopy (SEM) images and X-ray diffraction patterns (XRD). The magnetic response of PNCs was studied by means of a Vibrating Sample Magnetometer (VSM) at ambient temperature. In all cases magnetic phase content was used as a parameter.

## 2. Materials and Methods

The matrix of the prepared specimens was a two-component epoxy system. An epoxy prepolymer (diglycidyl ether of bisphenol-A) with the trade name Epoxol 2004 A and a curing reactant (aromatic diamine) with the trade name Epoxol 2004 B, both supplied by Neotex S.A. (Athens, Greece), were used for the synthesis of the polymer matrix. Iron oxide nanoparticles with a diameter less than 50 nm, zinc ferrite and strontium ferrite nanoparticles with diameters less than 100 nm, and barium titanate particles with a diameter less than 2 μm were used as the reinforcing phases. All ceramic particles were purchased by Sigma-Aldrich (Darmstadt, Germany).

**Figure 1:**
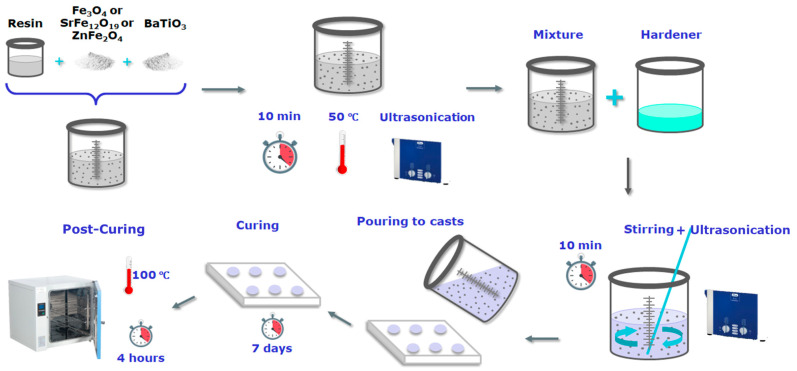
Schematic representation of the PNCs’ synthesis/fabrication procedure.

Each set of hybrid nanocomposites was constituent by the matrix, BaTO_3_, and a type of magnetic nanoparticles (Fe_3_O_4_, or ZnFe_2_O_4_, or SrFe_12_O_19_). Initially pre-calculated amounts of each type of MNPs and barium titanate were dispersed into the epoxy prepolymer at room temperature. The produced mixture was stirred in a sonicator (Elma S30H, Elmasonic, Singen, Germany) for ten minutes at 50 °C. Thus, a smooth dispersion of the reinforcing particles was achieved, and the formation of agglomerates was avoided. Then the mixture was left to reach ambient temperature. Afterwards the hardener (curing reactant) was added. The epoxy prepolymer and hardener were mixed at a 2:1 *w*/*w* ratio. The new mixture was exerted to sonication for another ten minutes at room temperature. In the next step, the hybrid mixture was put in molds made of silicone. Molds had geometries suitable for each of the experimental techniques used. Liquid mixtures were left in molds for polymerization/solidification at ambient temperature for a week. After the curing procedure was completed, samples were subjected to post-curing for four hours at 120 °C. It should be mentioned that in the case of ZnFe_2_O_4_, the specimen with 50 phr (parts per hundred resin per mass) was not examined further because difficulties occurred during its preparation. These difficulties, despite successive efforts, lead to poor mixing and the formation of extended agglomerations; thus this system was considered as not reliable. Figure 1 depicts a schematic representation of the PNCs’ synthesis/fabrication procedure.

In all series the content of barium titanate particles was constant at 10 phr (parts per hundred resin per mass), while the MNPs content varied at 5, 10, 15, 20, 40, and 50 phr. Table 1 lists the contents of the reinforcing phase for all fabricated hybrid systems.

SEM images were employed for examining the morphology of the fabricated hybrid PNCs and the quality of the nanoparticles’ dispersion in the epoxy resin. Images were obtained via a Carl Zeiss EVO MA 10 apparatus (Oberkochen, Germany).

XRD patterns from all four constituent materials and the produced hybrid PNCs were taken for structural characterization of all studied systems. The XRD patterns were obtained by means of a Bruker AXS D8 Advance (Coventry, UK) device with Bragg–Brentano geometry. The employed detector and the incident radiation spectral line were LynxEye and Cu Kα (*λ* = 1.54062 Å), respectively. The scan mode was continuous, with a 0.02° 2θ step and 0.5 s/step scan speed. The source slit was 0.6 mm while the voltage and current were at 40 kV and 40 mA, respectively.

The magnetic properties of the synthesized PNCs were tested and studied by employing a Vibrating Sample Magnetometer (VSM, Applied Research, Princeton, NJ, USA) at ambient temperature. The intensity of the applied magnetic fields varied from −20 to 20 kOe.

## 3. Results

### 3.1. Morphological/Structural Characterization

**Figure 2:**
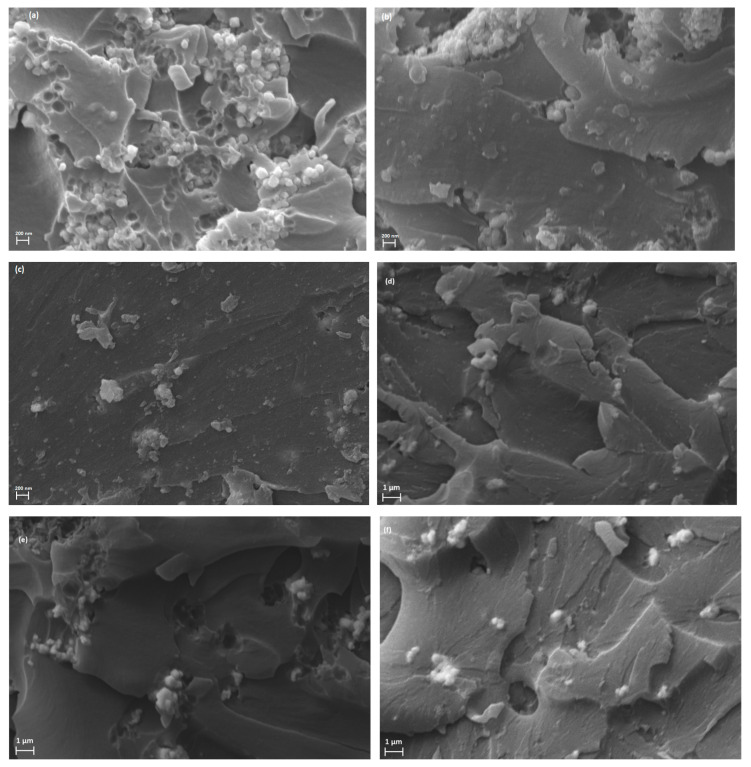
SEM images from the hybrid composites, 40 phr x/10 phr BaTiO_3_/epoxy, where x stands for (**a**) Fe_3_O_4_, (**b**) SrFe_12_O_19_, and (**c**) ZnFe_2_O_4_ at a high magnification, and SEM images from the hybrid composites, 10 phr x/10 phr BaTiO_3_/epoxy, where x stands for (**d**) Fe_3_O_4_, (**e**) SrFe_12_O_19_, and (**f**) ZnFe_2_O_4_ at a low magnification.

Figure 2 provides representative SEM images from all three sets of hybrid PNCs. Figure 2a–c present images of the nanocomposites with high filler content (40 phr x/10 phr BaTiO_3_/epoxy, where x stands for Fe_3_O_4,_ or, SrFe_12_O_19_, or ZnFe_2_O_4_) at a high magnification (65,000×), while Figure 2d,e depict images from systems with lower filler content (10 phr x/10 phr BaTiO_3_/epoxy, where x stands for Fe_3_O_4,_ or, SrFe_12_O_19_, or ZnFe_2_O_4_) at a lower magnification (15,000×). Cryo-fractured surfaces are indicative of the fine dispersion of the ceramic particles in the epoxy matrix since nanodispersions are evident in all cases in the absence of extensive clusters and voids. Limited agglomerations can be detected in specimens with the highest filler content. Employed nano- and micro-particles were used as received, without any surface modification. The occurring interactions within the hybrid PNCs are (i) between macromolecules and filler particles, and (ii) between the same or different type of filler particles. As the reinforcing phases increase, the second kind of interactions seems to prevail, thus forming limited clusters.

**Figure 3:**
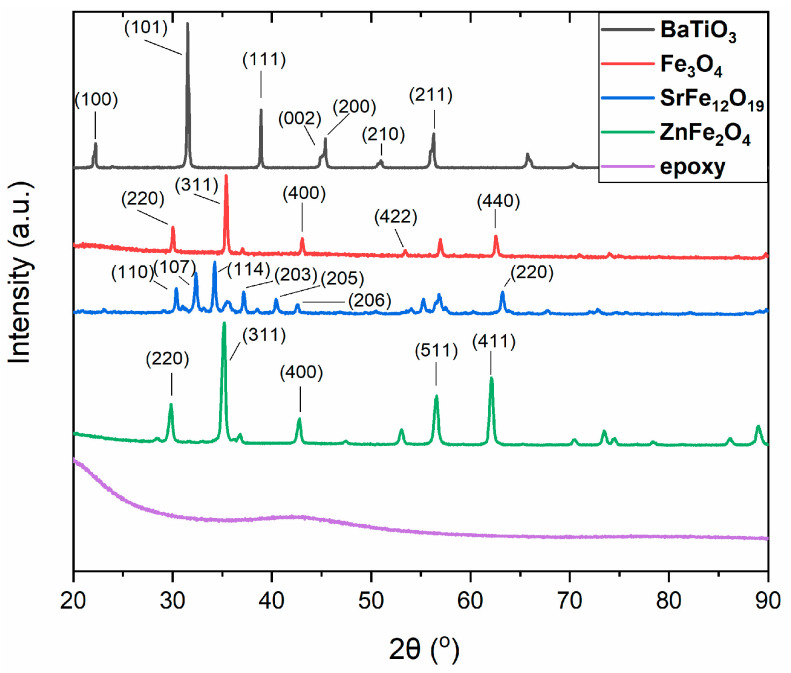
XRD patterns of the constituents’ materials.

**Figure 4:**
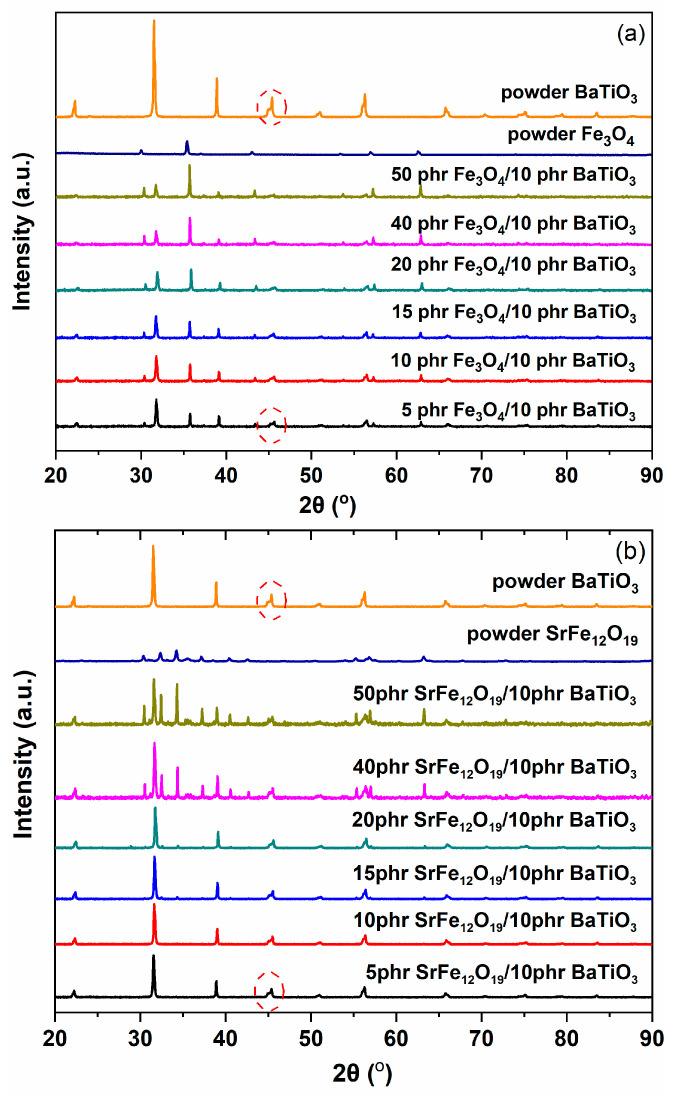

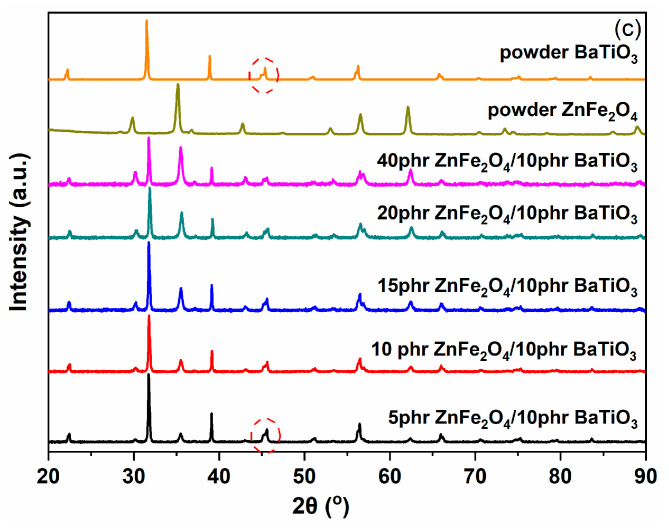
XRD patterns of the (**a**) Fe_3_O_4_/BaTiO_3_/epoxy system, (**b**) SrFe_12_O_19_/BaTiO_3_/epoxy system, and (**c**) ZnFe_2_O_4_/BaTiO_3_/epoxy system with varying magnetic phase content. Barium titanate’s (002) and (200) planes are indicated in the red dashed circle.

Figure 3 provides the XRD patterns of all the constituent materials of the synthesized hybrid PNCs. As expected, the amorphous thermosetting matrix exhibits no diffraction peaks. In the barium titanate diffractogram the characteristic planes (100), (101), (111), (002), (200), (210), and (211) are present. These planes correspond to the peaks that occur at 22.2, 31.5, 38.9, 44.9, 45.4, 51.0, and 56.3° of the 2θ, respectively. Interestingly the characteristic peaks (002) and (200) at 44.9 and 45.4°, considered as the “fingerprint” of the pure tetragonal phase [22,23,24,25,26,27], are detected. As can be seen in Figure 4, these peaks are also present in all patterns of the hybrid PNCs, indicating that the employed BaTiO_3_ particles are functional in switching between ferroelectric and paraelectric phases in all cases. At temperatures higher than T_C_, in the paraelectric phase, only the (200) diffraction peak occurs [22,23,24,25,26,27]. In the XRD pattern of magnetite, the recorded peaks at 30.0, 35.4, 43.1, 53.5, and 62.6° correspond to the planes (220), (311), (400), (422), and (440) [17,46,47,48]. In Figure 4 the peaks at 30.4, 32.4, 34.2, 37.1, 40.4, 42.6, and 63.2° of 2θ, corresponding to the (110), (107), (114), (203), (205), (206), and (220) planes, respectively, are characteristic of SrFe_12_O_19_ particles, giving evidence for a hexagonal crystal structure [46]. Furthermore, the main peaks of ZnFe_2_O_4_ in the XRD pattern are at 29.9, 35.2, 42.8, 56.5, and 62.1°, which correspond to the (220), (311), (400), (511), and (440) planes [46].

In Figure 4a–c, the XRD patterns of the three sets of hybrid PNCs can be seen. The diffractograms of the hybrid systems include peaks related to the employed reinforcing phases, implying their successful synthesis. Evidently, the intensity of the peaks increases relative to the incorporated amount of each nanofiller.

### 3.2. Magnetic Performance

**Figure 5:**
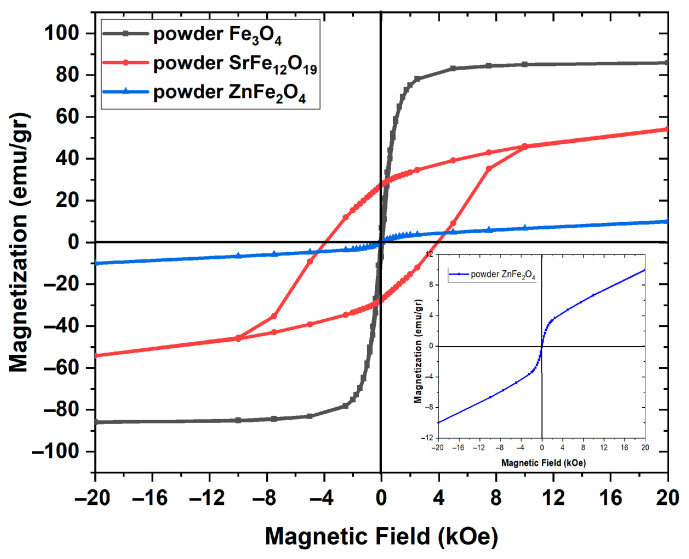
Magnetization curves of the three employed magnetic materials. The inset provides an enlargement of the magnetization curve of the ZnFe_2_O_4_ nanoparticles.

The hysteresis curves of magnetization versus the magnetic field’s intensity for the employed three magnetic nanopowders at ambient temperature are depicted in Figure 5.

The spectrum of Fe_3_O_4_ indicates soft ferrimagnetic behavior, being in accordance with the nanodimensions of the particles [17,33,37]. Magnetization acquires saturation rapidly at a field much lower than the highest one, taking a value close to 84 emu/gr. The very narrow hysteresis curve results in a low coercive field with an approximate value of 73 Oe. The hysteresis curves of SrFe_12_O_19_ imply a hard ferrimagnetic response [49]. The observed coercive field is about 4.0 kOe, and the recorded saturation approaches 50 emu/gr. In the spectrum of the magnetic curve of ZnFe_2_O_4_, an absence of coercivity and remanence magnetization can be observed. A negligible coercive field of ~22 Oe is recorded in Figure 5. In addition, magnetization seems not to achieve saturation even in the higher applied fields. The latter becomes more apparent in the inset of Figure 5. It should be [14,43,50,51,52] noted that an absence of saturation has been observed by applying a magnetic field up to 50 kOe [43]. This behavior leads to the conclusion that the employed zinc ferrite nanoparticles at room temperature are at a superparamagnetic state [14,43,50,51,52].

**Figure 6:**
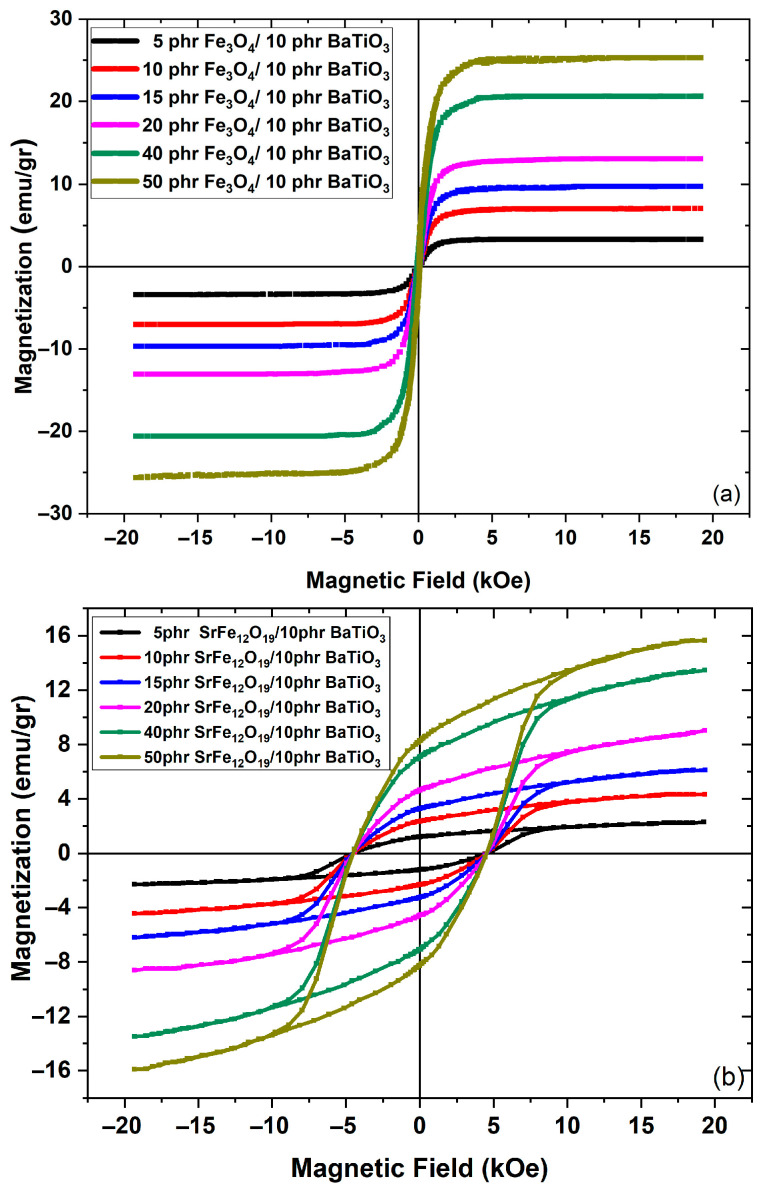

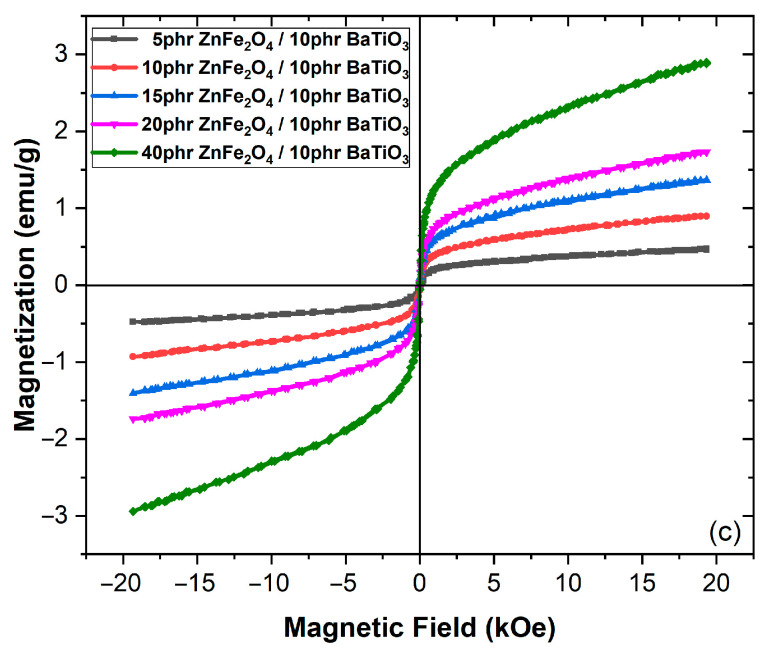
Magnetization curves of the hybrid PNCs with (**a**) Fe_3_O_4_, (**b**) SrFe_12_O_19_, and (**c**) ZnFe_2_O_4_.

The magnetic hysteresis loops of the nanocomposites are presented in Figure 6. It is apparent that magnetic nanoparticles induce magnetic properties to the nanocomposites, which resemble each time the performance of the specific magnetic phase. In all cases the magnetic response of PNCs is lower than the corresponding one of the pure magnetic phases because of the presence of two non-magnetic constituents (i.e., epoxy matrix and BaTiO_3_). The recorded magnetic response is enhanced with the increase in magnetic phase content. The soft ferrimagnetic behavior of magnetite is evident in Figure 6a, while hard ferrimagnetic and superparamagnetic responses are shown in Figure 6b,c for strontium ferrite and zinc ferrite, respectively. The coercive field of the PNCs with SrFe_12_O_19_ (Figure 6b) remains unaffected by the magnetic phase concentration since it is considered as an intrinsic property of the magnetic nanoparticles [53]. Previous studies of Fe_3_O_4_/epoxy and SrFe_12_O_19_/PLA composites revealed the same type of soft and hard magnetic behavior, respectively [16,54,55], although achieving lower saturation magnetization at a comparable magnetic phase content. This result could be attributed to the size and fine distribution of the employed nanoparticles.

## 4. Discussion

**Figure 7:**
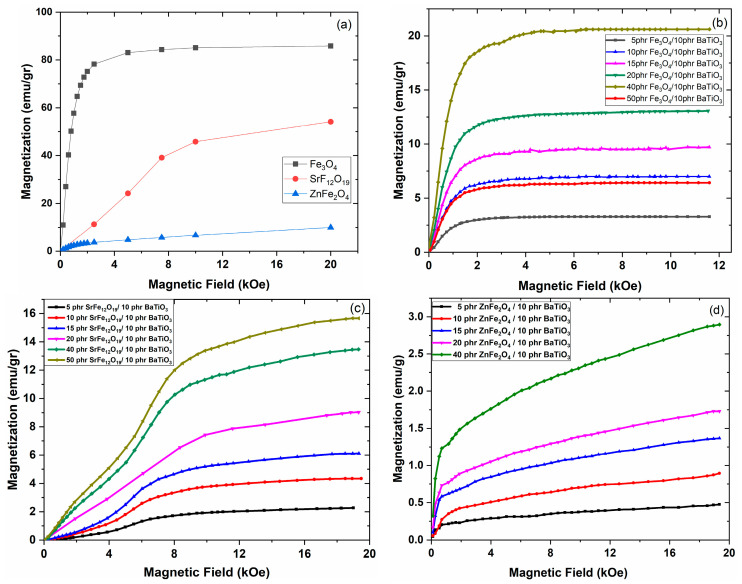
Initial magnetization curves for (**a**) pure magnetic nanopowders, (**b**) hybrid PNCs with Fe_3_O_4_, (**c**) hybrid PNCs with SrFe_12_O_19_, and (**d**) hybrid PNCs with ZnFe_2_O_4_.

The initial magnetization curves (also known as “virgin curves”) of the employed three magnetic phases can be seen in Figure 7a. Initial curves give the opportunity to determine magnetic susceptibility via the slope of the *M* = f(*H*) curves. At a low applied field, the relationship between magnetization and the applied field can be considered linear, and the obtained slope directly leads to magnetic susceptibility. At higher fields, where the curves sufficiently deviate from the linear relation, magnetic susceptibility curves can be evaluated according to Equation (1):

(1) $$x=\frac{\partial M}{\partial H}$$

From the initial linear part of the curves of Figure 7a and by employing Equation (1), the following mass magnetic susceptibility values, 7.524 × 10^−4^ m^3^/kg, 6.544 × 10^−5^ m^3^/kg, and 3.88 × 10^−5^ m^3^/kg, have been determined for the Fe_3_O_4_, SrFe_12_O_19_, and ZnFe_2_O_4_ nanopowders, respectively. It should be noted that by multiplying mass magnetic susceptibility with density, the unitless volume magnetic susceptibility can be obtained. Values of magnetic susceptibility vary with the particles’ dimensions and polycrystalline structure. The determined values lie within the limits reported in the literature [56,57,58,59]. Figure 7b–d present the initial curves for the different types of magnetic PNCs. Once again by employing Equation (1), at the initial linear part of the curves, the mass magnetic susceptibility for each nanocomposite system has been calculated. Determined values for mass magnetic susceptibility are listed in Table 1. As expected, magnetic susceptibility increases with magnetic phase content.

**Figure 8:**
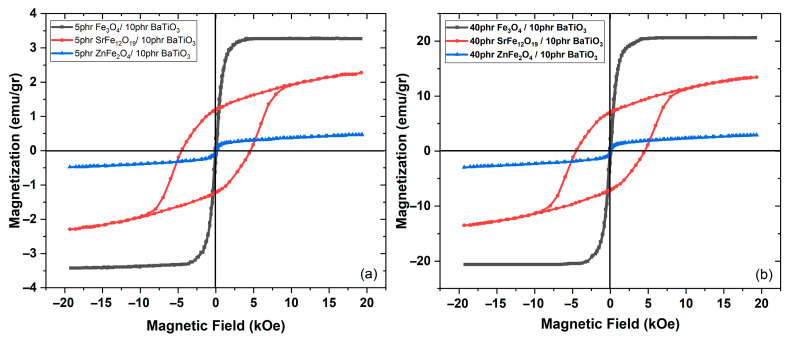
Comparative magnetization curves for the hybrid PNCs with varying of the type of magnetic phase at (**a**) 5 phr and (**b**) 40 phr magnetic phase content.

In Figure 8a,b a comparison of the recorded magnetization curves for the three types of magnetic PNCs is provided at low (Figure 8a) and high (Figure 8b) magnetic phase content. As can be seen, the magnetic performance of the hybrid PNCs is determined by the employed, in each case magnetic phase, and magnetization increase with magnetic filler content.

**Figure 9:**
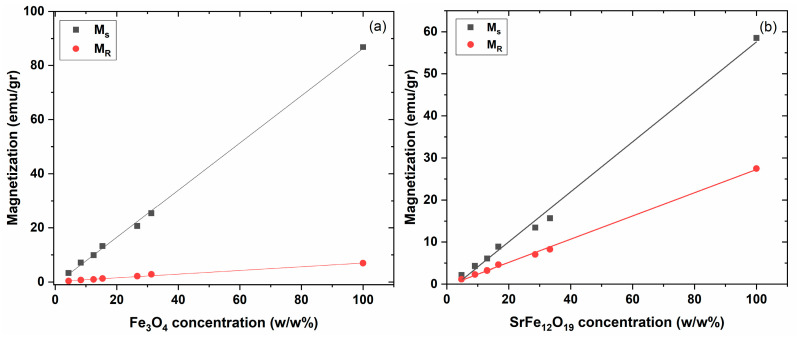
Magnetic saturation and magnetic remanence as a function of (**a**) Fe_3_O_4_ and (**b**) SrFe_12_O_19_ content.

Figure 9a,b provides plots of the magnetic saturation (*M_s_*) and magnetic remanence (*M_r_*) of the PNCs versus the magnetic filler content for the systems with Fe_3_O_4_ and SrFe_12_O_19_. The absence of saturation in the curves of ZnFe_2_O_4_, due to its superparamagnetic behavior, did not allow the formation of the respective graph. Interestingly, a linear relation between *M_s_*, *M_r_*, and magnetic phase content is established. A linear relation, up to 100% *w*/*w*, with magnetic phase content is an effective tool for adjusting the magnetic performance of PNCs according to the application’s requirements. In addition, this linear relation is a strong indication for the fine dispersion of the magnetic nanopowders [14,60].

## 5. Conclusions

In this study, three sets of epoxy matrix hybrid PNCs with magnetic inclusions were synthesized and experimentally tested. The employed magnetic nanoparticles were Fe_3_O_4_, or SrFe_12_O_19_, or ZnFe_2_O_4_ and varied in their content. In all hybrid PNCs a constant content of 10 phr of BaTiO_3_ microparticles was also added. The presence of barium titanate imparts functional behavior with respect to variable polarization and dielectric response because of the ferroelectric to paraelectric transition that it undergoes. The effect of barium titanate has been discussed elsewhere [4,5]. The successful synthesis of the hybrid PNCs was confirmed via SEM images and XRD patterns. The magnetic response of the fabricated systems and the pure magnetic phases were studied by means of magnetization curves as a function of the applied magnetic field, with the parameter being the magnetic phase content. Magnetization curves were recorded at ambient temperature via VSM. The type of magnetic behavior (i.e., soft, hard, or superparamagnetic) exhibited by Fe_3_O_4_, SrFe_12_O_19_, and ZnFe_2_O_4_, respectively, was induced in hybrid PNCs. Magnetic performance was altered with magnetic phase content in all cases. Initial magnetization curves were employed for the determination of mass magnetic susceptibility of each nanosystem. Values of susceptibility, approximated by the initial linear part of the “virgin” curves, increase with magnetic phase content. Magnetic saturation (*M_s_*) and magnetic remanence (*M_r_*) follow a linear relation with magnetic phase content, which could prove useful in predicting magnetic performance in applications.

## Figures and Tables

**Table 1:** Filler content and mass magnetic susceptibility (*x*) for the three series of hybrid PNCs.

Fe_3_O_4_/BaTiO_3_		SrFe_12_O_19_/BaTiO_3_		ZnFe_2_O_4_/BaTiO_3_	
Content (phr)	*x* (m^3^/kg)	Content (phr)	*x* (m^3^/kg)	Content (phr)	*x* (m^3^/kg)
Neat epoxy	-	Neat epoxy	-	Neat epoxy	-
5/10	2.864 × 10^−5^	5/10	2.126 × 10^−6^	5/10	2.022 × 10^−6^
10/10	6.104 × 10^−5^	10/10	4.082 × 10^−6^	10/10	6.580 × 10^−6^
15/10	9.269 × 10^−5^	15/10	4.283 × 10^−6^	15/10	6.720 × 10^−6^
20/10	1.226 × 10^−4^	20/10	9.809 × 10^−6^	20/10	7.342 × 10^−6^
40/10	1.966 × 10^−4^	40/10	1.557 × 10^−5^	40/10	1.216 × 10^−5^
50/10	1.997 × 10^−4^	50/10	1.696 × 10^−5^	-	-

## Data Availability

Data are available upon reasonable request.
